# Physical Demands and Acute Neuromuscular Responses Across a Single-Day 3 × 3 Male Basketball Tournament

**DOI:** 10.3390/s25113296

**Published:** 2025-05-23

**Authors:** Pierpaolo Sansone, Vincenzo Rago, Enrique Alonso-Perez-Chao, Shaoliang Zhang, Daniele Conte

**Affiliations:** 1 Department of Education and Sport Sciences, Pegaso Telematic University, Centro Direzionale Isola F2, 80143 Naples, Italy; 2 National Youth Sports Institute, Singapore 397778, Singapore; vincenzo.rago@live.com; 3 Faculty of Sports Sciences, Universidad Alfonso X el Sabio, 28691 Villanueva de la Cañada, Spain; eperezch@uax.es; 4 Faculty of Sports Science, Universidad Europea de Madrid, 28001 Villaviciosa de Odón, Spain; 5 Athletic Performance and Data Science Laboratory, Division of Sports Science and Physical Education, Tsinghua University, Beijing 100084, China; zslinef@mail.tsinghua.edu.cn; 6 Department of Movement, Human and Health Sciences, University of Rome “Foro Italico”, 00135 Rome, Italy; daniele.conte@uniroma4.it

**Keywords:** athlete monitoring, microsensors, fatigue, team sports, Olympic sport, CMJ, physical demands, three-on-three

## Abstract

**Background**: This study examined external intensity and acute neuromuscular responses across multiple games played during a single-day official 3 × 3 basketball tournament. **Methods**: Twelve male players (Tier 2–3; age: 24.7 ± 4.5 years; height: 186.4 ± 8.5 cm; body mass: 86.5 ± 13.0 kg) were monitored with microsensors (Movement Intensity (MI), while countermovement jump (CMJ) variables—jump height (JH); time to takeoff (TTTO); and Modified Reactive Strength Index (RSI_mod_)—were obtained before the start of the tournament and after each game. Linear mixed models examined differences in MI and CMJ variables across tournament phases. Additionally, the smallest worthwhile change (SWC) calculations were applied to all comparisons. **Results**: No statistical differences were found across tournament stages for MI (*p* = 0.466), JH (*p* = 0.762), TTTO (*p* = 0.990), or RSI_mod_ (*p* = 0.951). SWC comparisons showed that MI was higher in GG1 than GG2, GG3, and QF; higher in GG2 than GG3; and lower in G3 than QF and SF. Regarding JH, the post-QF value was higher than the baseline and post-GG2. For TTTO, post-QF was higher than post-GG1. RSI_mod_ post-GG2 was lower than post-GG3 and post-SF. **Conclusions**: While no significant changes were observed, MI showed a practically meaningful decline in GG3 and recovery in QF, while RSI_mod_ initially declined before improving post-SF. These findings highlight the importance of pacing and recovery strategies in 3 × 3 basketball tournaments.

## 1. Introduction

The game of 3 × 3 basketball is a rapidly growing team sport that has gained significant attention from fans and sport scientists, particularly after its inclusion in the last two Olympic games (Tokyo 2020 and Paris 2024), with its presence confirmed for Los Angeles 2028. This prestigious recognition follows the sport’s steady development, with 182 national federations currently competing in official national, continental, and international (world) competitions organized by the 3 × 3 International Basketball Federation (FIBA).

A recent systematic review [1] revealed that 3 × 3 basketball presents intermittent demands, characterized by a fast pace and short ball possessions (6–8 s), accumulating 62–94 changes in direction, 17–33 accelerations, 24–44 decelerations, and 17–24 jumps per game [1]. A key characteristic of 3 × 3 basketball is its unique competition format, consisting of a group stage followed by a knockout stage, requiring teams to play multiple games (n = 2–3) per day over consecutive days. Consequently, players might accumulate substantial external loads with non-sufficient recovery time, leading to fatigue [2] and associated performance impairments [3]. Evidence from the FIBA 2019 World Cup [4] showed that players spent less time performing high-intensity activities and more time recovering during the knockout stage than the group stages, potentially indicating signs of neuromuscular fatigue as the tournament progressed. However, no study has directly assessed acute neuromuscular responses during single-day 3 × 3 basketball tournaments. Furthermore, existing research has yet to establish a consensus on whether external load intensity fluctuates across different tournament stages.

According to the external–internal load model [5], the response of an athlete’s biological systems, and, thus, their acute status (i.e., readiness) are dependent on the dose (i.e., external load) imposed by training and competition. Thus, it is crucial to understand how the external loads encountered in 3 × 3 basketball tournaments, which require multiple games on the same day, influence players’ readiness and physical performances. Given the high neuromuscular demands of this sport [1] and the congested competition schedule, it would be useful for practitioners to determine whether 3 × 3 basketball players experience neuromuscular fatigue during different phases of a tournament. This is particularly relevant, as fatigue has been shown to negatively impact basketball players’ physical [2] and technical–tactical [6,7] performances, potentially affecting their team’s overall success. Among the available methodologies for assessing the athletes’ neuromuscular status, countermovement jump (CMJ) testing is one of the most widely implemented in applied sport science thanks to its relevance for athletic performance [8], its validity and reliability [9,10], and the short time necessary for testing procedures. Therefore, the aim of this study was to monitor the external intensity outputs alongside neuromuscular status (monitored using CMJ) during an official single-day 3 × 3 male basketball tournament. We hypothesize that external load metrics are expected to progressively decrease throughout the tournament, while neuromuscular fatigue markers are anticipated to show signs of impairment, particularly in later matches, due to the high physical demands and limited recovery time inherent to the 3 × 3 basketball competition format.

## 2. Materials and Methods

### 2.1. Participants and Design

This observational, repeated-measures study monitored 12 male athletes (age: 24.7 ± 4.5 years; height: 186.4 ± 8.5 cm; body mass: 86.5 ± 13.0 kg) belonging to 3 different 3 × 3 basketball teams during a single-day official tournament. The tournament took place on a single day, with all games played outdoors. The recruited players regularly competed in 5 vs. 5 basketball leagues at the regional level, demonstrating proficiency in sport-specific skills (tier 2–3) [11]. Prior to participation, all players were informed about the monitoring procedures and provided written informed consent. The study was approved by the Institutional Review Board of the University of Rome “Foro Italico” (approval number: CAR 208/2024). No injuries were reported during the monitored tournament.

### 2.2. Tournament Structure

During the tournament, each team played three group-stage games—group game 1 (GG1), group game 2 (GG2), and group game 3 (GG3)—with 30 min breaks between games. Following the group stage, there was a 60 min break, after which the team that qualified for the elimination round played the quarterfinals (QF), semifinals (SF), and final (F), if they advanced. Knockout-stage games were also interspersed by 30 min breaks. However, no data from the final match were collected, as none of the monitored teams reached this stage.

All games were played following the official FIBA 3 × 3 basketball rules. Matches were played on a 15 × 11 m (length × width) court with a single hoop, lasting 10 min or ending earlier if a team reached 21 points. Teams were required to attempt a shot within 12 s of gaining possession. The scoring system awarded 1 point for successful shots inside the arc or free throws, and 2 points for shots made from beyond the arc.

### 2.3. External Intensity Monitoring

Before the start of the first game, players were equipped with Firstbeat Sports devices (Firstbeat; Jyväskylä, Finland), which comprise a lightweight, water and shock-proof 9-axis motion sensor (accelerometer, gyroscope, and magnetometer). The devices were firmly affixed to the players’ chests, and worn under the players’ sports jerseys at the base of the sternum via textile straps, which were tightened as much as possible to reduce motion artifacts and increase the accuracy of the accelerometry data. The textile straps also feature a Movesense connector to which the motion sensor is clipped, securing it to the athlete’s body and reducing data error further. The devices’ reliability has been previously demonstrated [12], with a recent study also implementing them to monitor the physical demands of 3 × 3 basketball settings [13]. The Movement Intensity (MI) variable was calculated by dividing the sum of accelerations performed across the three movement axes by the game duration (in minutes). This metric represents the external intensity imposed on the athlete’s body. MI has been previously shown to discriminate external intensities between basketball players of different competitive levels [14].

### 2.4. Neuromuscular Status

To monitor the players’ neuromuscular status, a bilateral, no-arm-swing countermovement jump (CMJ) was implemented. CMJ testing provides valid and reliable insights into an athlete’s neuromuscular status [10] and has been widely used in team sports due to its involvement in the stretch-shortening cycle [8,15], making it particularly relevant for 3 × 3 basketball. CMJs were assessed at baseline (prior to the first game of the tournament) and immediately after each completed game.

Before the baseline CMJ trial, athletes completed an 8 min standardized warm-up consisting of jogging, dynamic stretching, squats, lunges, plyometrics, and three submaximal-to-maximal CMJ trials. In contrast, subsequent CMJ assessments were conducted without a warm-up, with players allowed a brief recovery period (<2 min) to rehydrate and recover.

CMJs were performed as follows [9,16]: athletes began in an upright, comfortable position, with their legs fully extended, feet positioned at a self-selected width, and hands resting on their hips. They then executed a countermovement to their preferred depth before performing a maximal vertical jump. Athletes were instructed to exert maximal effort and aim for the highest possible jump. To minimize measurement error, two CMJ trials were conducted at each time point, with a 1 min rest between trials [9,16]. The best performance value among the two trials was considered for analyses to mitigate any confounding learning effects among players with varying CMJ experience [17].

The *MyJump Lab* smartphone application was used to analyze CMJ performance. This application has been demonstrated to be valid and reliable in assessing the CMJ metrics selected for this research [9,16,18]: jump height (JH; vertical displacement of the athlete’s center of mass, in cm); time to take-off (time, in s, between the initiation of the countermovement to the moment of take-off, TTTO), and Modified Reactive Strength Index (RSI_mod_, the ratio between JH and TTTO, in AU). These variables were chosen for analyses since JH represents the performance proxy measure, while TTTO and RSI_mod_ express changes in the neuromuscular strategy of athletes, which are indicative of the athlete’s neuromuscular status and fatigue [10]. Specifically, higher JH represents better overall performance, while shorter TTTO alongside maintained JH and higher RSI_mod_ indicate better neuromuscular status [10]. JH, TTTO, and RSI_mod_ obtained with *MyJump Lab* are highly accurate, comparable to those measured with gold-standard instruments (e.g., 1000 Hz force plates), demonstrating good to excellent reliability (intraclass correlation coefficient: 0.79–0.94) [9,19]. An experienced sport scientist (>5 years of experience) proficient in the MyJump Lab application recorded and analyzed all CMJ trials. Videos were captured at 240 fps using an Apple iPhone 12 (Apple, Cupertino, CA, USA), ensuring the athlete’s full body remained within the frame throughout the ground contact phase. The videos were taken in proper lighting conditions, with excessive background light avoided. Dark conditions were not applicable, as the tournaments took place during the day and the court lights were turned on when the sun set. Neuromuscular fatigue was identified as follows [2,10]: an increase in TTTO without a concurrent increase in JH compared to the previous time point and/or a reduction in RSI_mod_ relative to the previous measurement.

### 2.5. Statistical Analyses

Data analyses were performed using *Jamovi* (Jamovi project) statistical software (version 2.3) and customized Excel spreadsheets, with the α value set at 0.05. Linear mixed models were performed to evaluate external load intensity (MI) and CMJ variables (JH, TTTO, RSI_mod_) across the different tournament phases. The game (five levels: GG1, GG2, GG3, QF, SF) was inserted in the models as fixed effects, whereas players’ id was inserted as random effects to account for repeated measures. Post-hoc Bonferroni tests were implemented to identify changes between games for MI and CMJ variables. Akaike’s Information Criterion (AIC), F, *p* values, and estimated marginal means (EMM) (mean ± standard error [SE]) were reported for all models. For pairwise comparisons, effect sizes (ES) were calculated as Hedges’ g [20], and interpreted as follows: <0.20, trivial; 0.20 to 0.59, small; 0.60 to 1.19, moderate; 1.20 to 1.99, large; and >2.0, very large.

Additionally, the smallest worthwhile change (SWC) was calculated to identify practically significant changes in physical demands and CMJ variables [21,22]. For each variable, the SWC was calculated by multiplying the between-group standard deviation by 0.2 [20,22], as this threshold permits the identification of changes that are more than trivial across homogeneous samples (i.e., same competitive level), and is thus practically interesting [23]. Then, the absolute difference in each pairwise comparison obtained from the mixed models was compared to the SWC obtained to identify practically meaningful changes (>SWC) in external load intensity and neuromuscular responses across tournament phases.

## 3. Results

Table 1 reports the average MI values for each player. Inter-trial variability for the CMJ measures was good to excellent (intraclass correlation coefficients: 0.78–0.93). Table 2 presents the mixed model results for MI, JH, TTTO, and RSI_mod_, with no effect of the tournament stage on either external load intensity or CMJ variables.

**Figure 1:**
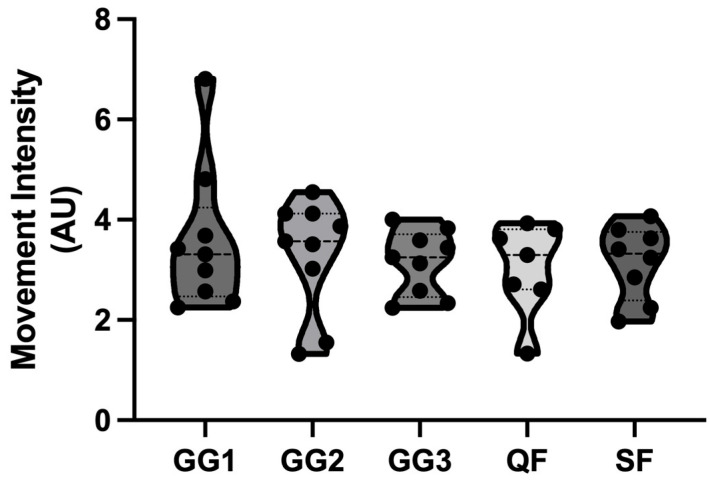
Movement Intensity across tournament phases. GG1: group game 1; GG2: group game 2; GG3: group game 3; QF: quarterfinals; SF: semifinals.

**Figure 2:**
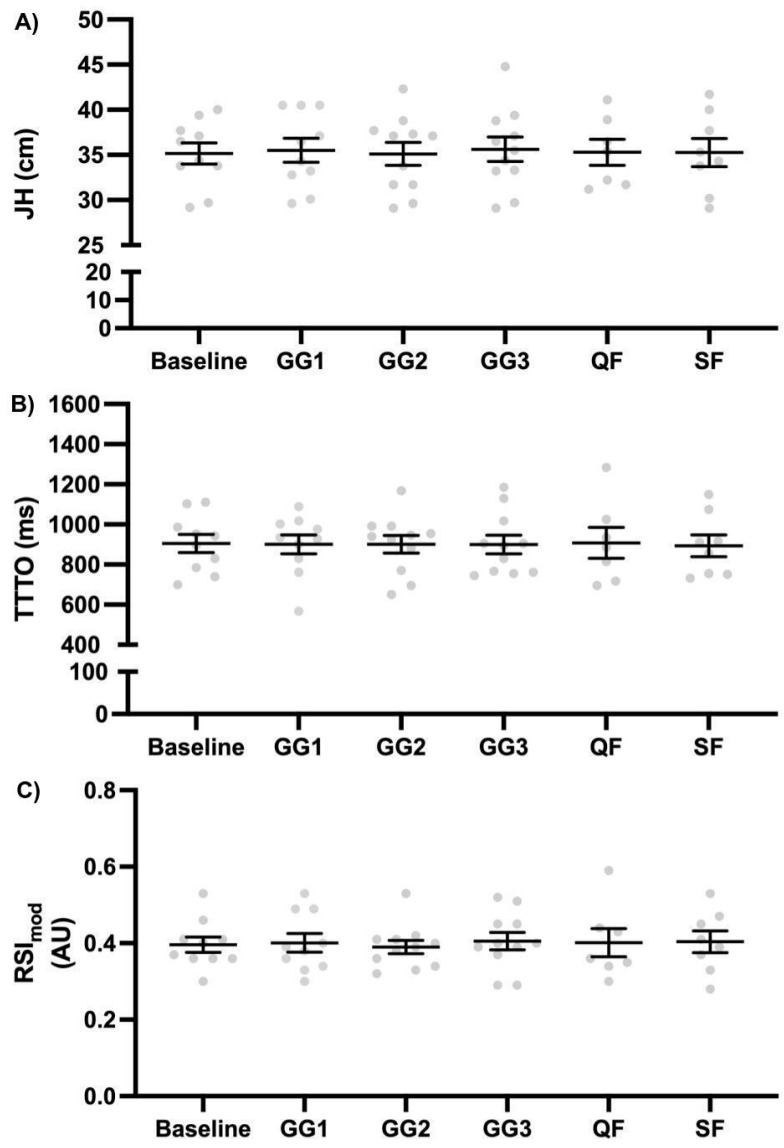
Countermovement jump responses across tournament phases. (**A**) JH: jump height; (**B**) TTTO: time to take-off; (**C**) RSI_mod_: Modified Reactive Strength Index. GG1: group game 1; GG2: group game 2; GG3: group game 3; QF: quarterfinals; SF: semifinals.

The SWC thresholds for the dependent variables were as follows: MI, 0.20 AU; JH, 0.79 cm; TTTO, 29.73 ms; and RSI_mod_, 0.01 AU. Figure 1 and Figure 2 present results (mean and standard error) across tournament phases for MI and CMJ variables, respectively. Bonferroni tests showed no significant pairwise difference between tournament phases (all *p* > 0.05) for all variables. Regarding MI, GG3 tended to be lower than all other games, with small ES; in contrast, all other comparisons held only trivial ESs. For JH, TTTO and RSI_mod_, all comparisons were non-significant and held trivial ESs [23].

For practically meaningful results, considering SWC, MI was higher in GG1 than GG2, GG3, and QF; additionally, it was higher in GG2 than GG3, and lower in GG3 than QF and SF. Regarding JH, the post-QF value was higher than the baseline and post-GG2. For TTTO, post-QF was higher than post-GG1. RSI_mod_ post-GG2 was lower than post-GG3 and post-SF.

## 4. Discussion

The aim of this study was to monitor the external intensity outputs alongside neuromuscular fatigue markers during an official single-day 3 × 3 male basketball tournament. The main findings were as follows: (1) MI showed no statistically significant changes across tournament phases, (2) MI was highest in G1 and SF, while it was lowest in G3, (3) no statistically significant changes were observed in CMJ variables, and (4) a slight improvement in RSI_mod_ post-G3 and post-SF compared to post-G2.

The lack of statistically significant changes in MI across tournament phases contrasts with the findings of Willberg et al. [24], who initially suggested that external load parameters might decrease over the course of a tournament. However, their results showed that external intensity per minute was highest in some parameters (jumps and meters per minute) during the final games. In this regard, session-RPE values also progressively increased throughout the tournament in their study [24], indicating a rising perceived exertion. It is possible that 3 × 3 players strategically distribute their energy reserves, particularly in a single-day tournament format, where the ability to sustain performance across multiple games may be crucial. In addition, the rolling substitution rule may also allow players to maintain similar physical intensity levels throughout the competition.

The second key finding was that MI peaked in GG1 and SF, while it was lowest in GG3. This pattern suggests that players exert maximal effort during the initial game, likely due to their lack of accumulated fatigue. This pattern is similar to what most 5 vs. 5 basketball research has found regarding physical intensity across game phases, with most studies finding greater external intensity values in the first quarter of the game, when players are fresh and in their best conditions. In the current study, higher MI was also found in critical elimination rounds, aligning with findings by Ferioli et al. [25], who reported increased game intensity in high-stakes 3 × 3 basketball matches. Along these lines, it is possible that GG3 had lower intensity considering that some of the involved teams might have already been qualified or eliminated from the knockout stage due to their group ranking following previous games’ results.

No statistically significant changes were observed in any CMJ variables, suggesting that neuromuscular fatigue was not manifested throughout the tournament. This finding is consistent with the previous literature suggesting that short-term exposure to 3 × 3 basketball competitions does not necessarily induce significant decrements in physical performance [24]. The low-volume nature of 3 × 3 play, along with structured breaks, may allow players to recover sufficiently between games, thereby mitigating excessive declines in neuromuscular parameters [24]. These results reinforce the notion that neuromuscular fatigue in 3 × 3 basketball might be less pronounced than in traditional 5 vs. 5 formats [26], due to the major differences in single game volume.

Regarding the practically meaningful improvements in RSI_mod_ post-GG3 and post-SF compared to post-GG2, this finding suggests an adaptive response in stretch-shortening cycle efficiency. This phenomenon may be attributed to the potentiation effects of repeated high-intensity efforts, a response also noted in other 5 vs. 5 basketball [27]. However, these value changes were trivial in size, thus indicating that overall, players’ neuromuscular status was substantially unchanged across the single-day tournament. Such findings suggest that players might experience acute neuromuscular facilitation rather than pure fatigue during certain phases of competition, particularly when sufficient recovery breaks are allowed. In fact, in the current single-day tournament, players had a 1:3 gameplay/rest ratio across the tournament, plus an additional recovery break of 60 min between the group and the knockout phases, which altogether permitted players to sufficiently recover as seen from the absence of neuromuscular fatigue.

The limitations encountered in completing this study should be considered when interpreting our results. First, the sample size was relatively small, which may limit the generalizability of the findings. Additionally, the study focused exclusively on adult male players, excluding youth, female, and elite-level athletes, meaning that the results can only be applied to similar populations. Another key limitation is the specific context of the study—a single-day tournament. This format differs from multi-day 3 × 3 tournaments, where recovery periods between days might influence players’ physical and neuromuscular responses differently. Future research should explore these aspects in larger and more diverse samples, as well as in multi-day tournament settings, to provide a more comprehensive understanding of external intensity and neuromuscular fatigue in 3 × 3 basketball.

## 5. Conclusions

No significant tournament-induced changes were observed either for external intensity or CMJ variables, suggesting that players might have adjusted their pacing strategies to manage accumulated neuromuscular fatigue. Movement intensity declined during the late group stages and increased again during the knockout rounds, likely influenced by the importance of knockout games and the opportunity for recovery permitted by the between-games breaks. Furthermore, acute neuromuscular responses remained stable across the tournament. However, minor improvements in neuromuscular status were observed following the longer break before elimination rounds, suggesting that recovery periods of ~60 min may provide benefit in maintaining neuromuscular status. These findings highlight the importance of pacing and recovery strategies in optimizing performance during high-density 3 × 3 basketball tournaments. Given the study’s limitations, including a small sample size and focus on a single-day competition, future research should investigate the effects of multi-day tournaments and different recovery protocols to enhance player performance and fatigue management.

## Figures and Tables

**Table 1:** Individual player Movement Intensity values (mean and standard deviation).

	Movement Intensity
Player 1	2.63 ± 0.89
Player 2	5.40 ± 1.99
Player 3	4.27 ± 0.73
Player 4	3.19 ± 0.60
Player 5	1.72 ± 0.47
Player 6	3.46 ± 0.24
Player 7	3.19 ± 0.59
Player 8	3.55 ± 0.59
Player 9	3.17 ± 0.75
Player 10	2.85 ± 0.36
Player 11	3.44 ± 0.96
Player 12	2.28 ± 0.77

**Table 2:** Results of mixed models for the variables assessed.

Variable	AIC	F	*p*	Random-Effects Variance	Residual Variance
Movement Intensity	119.994	0.922	0.466	0.705	0.523
Jump Height	269.826	0.516	0.762	12.99	2.83
Time to Take-Off	725.31150	0.107	0.990	14,000	8761
Modified Reactive Strength Index	−159.883	0.223	0.951	0.003	0.001

## Data Availability

Data will be made available upon reasonable request.

## Abbreviations

The following abbreviations are used in this manuscript:

CMJ	Countermovement jump
ES	Effect size
GG1	Group game 1
GG2	Group game 2
GG3	Group game 3
JH	Jump height
MI	Movement Intensity
QF	Quarterfinals
RSI_mod_	Modified Reactive Strength Index
SF	Semifinals
SWC	Smallest worthwhile change
TTTO	Time to take-off
